# Development of a monoclonal antibody-based approach for selective enrichment of target *Bifidobacterium longum* from a complex fecal community

**DOI:** 10.1080/29933935.2026.2663732

**Published:** 2026-04-29

**Authors:** Gaku Nakato, Hikaru Inoue, Satoshi Onawa, Risako Furukawa, Nozomu Obana, Kazuki Tanaka, Hitosi Agematu, Isaiah Song, Joe Inoue, Shinji Fukuda

**Affiliations:** a Gut Environmental Design Group, Kanagawa Institute of Industrial Science and Technology, Kawasaki, Kanagawa, Japan; b Innovative Microbiome Therapy Research Center, Juntendo University Graduate School of Medicine, Tokyo, Japan; c Institute for Advanced Biosciences, Keio University, Tsuruoka, Yamagata, Japan; d Transborder Medical Research Center, Institute of Medicine, University of Tsukuba, Tsukuba, Ibaraki, Japan; e Division of Biochemistry, Faculty of Pharmacy and Graduate School of Pharmaceutical Sciences, Keio University, Tokyo, Japan

**Keywords:** Monoclonal antibody, *Bifidobacterium longum*, microbiome, selective enrichment, immunomagnetic separation

## Abstract

Individual differences in gut microbiota composition highlight the need for methods capable of selectively enriching host-associated bacteria from complex microbial communities. Conventional cultivation approaches lack the precision required for targeted enrichment, limiting progress in personalized microbiome research. Here, we established a proof-of-concept monoclonal antibody-based strategy for the selective enrichment of a target gut bacterium. We generated a monoclonal antibody (8H2) exhibiting preferential reactivity toward the human-derived *Bifidobacterium longum* Jih1 and demonstrated that it selectively enriched viable Jih1 cells from a defined bacterial consortium and a human fecal sample. Proteomic and genetic analyses suggested that 8H2 recognizes glutamine synthetase (GS), an enzyme typically localized intracellularly, but detected on the surface of Jih1 cells. This surface association enables antibody binding and facilitates selective enrichment within complex microbial communities. These data support the feasibility of antibody-based, selective enrichment of viable bacteria and suggest potential applications for monitoring individual-associated bacteria in personalized nutrition and microbiome-based interventions.

## Introduction

A large number of microbes colonize the human gut, totaling approximately 38 trillion bacterial cells.^1^ Despite continuous exposure to environmental and dietary perturbations, the gut microbiota generally maintains functional stability, a property often described as microbial robustness.^2^ However, the breakdown of gut microbial robustness can lead to the onset of gut-related diseases, including those of a metabolic or systemic nature.^3-5^ Altered gut microbial compositions relative to those of resident communities in healthy humans are broadly defined as dysbiosis.^6^ Various strategies have been proposed to restore microbial balance, including probiotic supplementation. However, sustained colonization and long-term maintenance of administered strains remain inconsistent.^7^^,^^8^ Increasing evidence indicates that colonization patterns vary substantially among individuals, suggesting that host-associated bacteria may contribute to personalized microbial stability and function.^9^ These findings highlight the potential relevance of host-associated bacteria in achieving individualized microbial stability.

In efforts to enrich host-associated bacteria, the conventional method of using selective media can be inefficient and time-consuming due to indiscriminate isolation of non-target species.^10^ Moreover, approximately one-third to two-thirds of gut bacterial taxa still lack cultured representatives,^11^ and many cultivation strategies depend on stochastic recovery of bacteria lacking clear phenotypic markers, making low-abundance or phenotypically indistinct bacteria particularly difficult to obtain.^12^^,^^13^ To overcome these limitations, several antibody-based bacterial enrichment methods have been developed.^14^^,^^15^ Antibodies can, in principle, capture target bacteria regardless of abundance or growth characteristics, and recent studies have demonstrated successful enrichment of uncultured species using antibody-mediated approaches.^15^ In 2023, a polyclonal antibody-coated immunomagnetic bead method enabled the enrichment and cultivation of *Fusobacterium nucleatum* from artificial fecal samples.^16^ However, polyclonal antibodies inherently suffer from limited antigen specificity and batch-to-batch variability, which may restrict their reproducibility and targeting performance.^17^ A later study reported a monoclonal antibody targeting *Bifidobacterium*, but the antibody displayed polyreactivity toward multiple species within the genus, underscoring the challenge of achieving highly selective enrichment.^10^ Collectively, these findings suggest that selective media and existing antibody-based methods remain limited in enabling reliable and reproducible enrichment of target bacteria within complex microbial communities, and that methodological refinement may improve the performance of monoclonal antibody-based enrichment approaches.

Here, we aimed to establish a monoclonal antibody-based selective enrichment method as a proof of concept. Compared to polyclonal antibodies, monoclonal antibodies against bacteria offer defined antigen-binding specificity, low background signal, and reproducible performance.^18^ Using the secondary lymphoid organ transplantation (SLOT) technique – a recently developed method for rapidly generating high-affinity monoclonal antibodies against complex microbial antigens – we generated a monoclonal antibody with preferential reactivity toward our target bacterium. We demonstrated this concept using *Bifidobacterium longum* Jih1, a well-studied human commensal bacterium with reported probiotic functions, including support of mucosal barrier integrity and mitigation of intestinal inflammation.^19^^,^^20^ Jih1 is a human-derived bacterium with a fully sequenced genome.^21^ As such, we used this bacterium to evaluate the feasibility and utility of a monoclonal antibody-based approach for selective enrichment within complex microbial communities.

## Results

### Evaluation of target bacterium *B. longum* Jih1

To select an appropriate model bacterium for monoclonal antibody development and selective enrichment evaluation, we first considered *Bifidobacterium longum*, a well-known probiotic bacterium. Effective probiotic function depends not only on bacterial viability but also on the ability to establish stable colonization and interact with the host immune system. During our initial characterization of the candidate bacterium, we observed that the human-derived *B. longum* Jih1 reproducibly outcompeted the JCM1217 strain in germ-free mice (Supplementary Figure 1A) and exhibited higher MV production under the tested conditions (Supplementary Figure 1B). Increasing evidence suggests that bacterial membrane vesicles (MVs) may contribute to host colonization processes, including host immune stimulation and microbial fitness.^22^^,^^23^ Altogether, based on its human origin, availability of a complete genome sequence, and suitability as an experimentally tractable model bacterium, Jih1 was selected as a model bacterium for evaluating the feasibility of SLOT-based monoclonal antibody generation and selective enrichment of target bacteria.

### Generation of monoclonal antibody against *B. longum* Jih1 using the SLOT method

**Figure 1.**
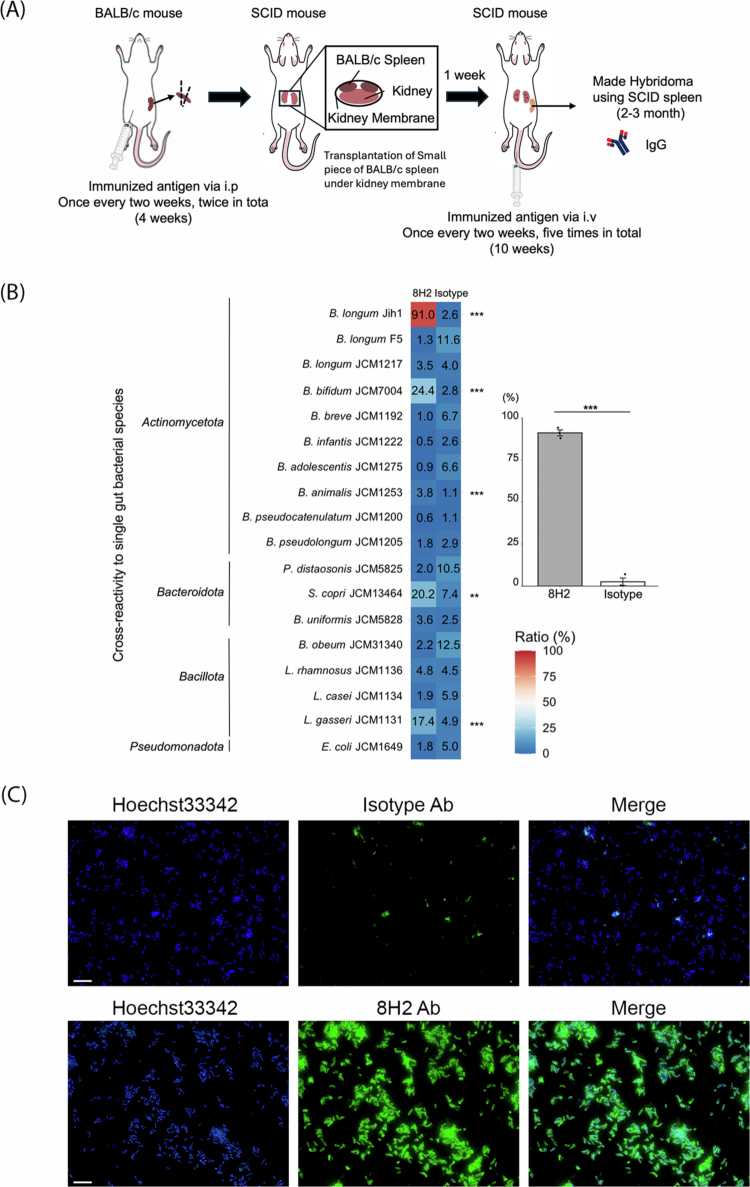
Generation of a monoclonal antibody reactive to human *B. longum* Jih1. (A) Experimental immunization scheme for producing a monoclonal antibody against the human *B. longum* Jih1 antibody. (B) Heat map showing 8H2 and isotype control IgG_3_ reactivity to various bacteria as evaluated by flow cytometry. Significance metrics for the reactivities of isotype IgG3κ and each bacterium were calculated using Welch's t-test. **P < 0.01, ***P < 0.001. The data represent the mean values from three technical replicates. (C) Immunofluorescence staining of Jih1 staining with 8H2 or isotype control antibody (green) and Hoechst 33342 (blue). Scale bars, 10 μm. Representative images from at least three independent experiments.

To generate monoclonal antibodies against Jih1, we used the secondary lymphoid organ transplantation (SLOT) method, an *in vivo* adaptation of a previously developed artificial lymph node-based antibody production system.^24^^,^^25^ First, BALB/c mice were immunized with Jih1 via intraperitoneal injection, and then the spleen of each mouse was transplanted into the renal subcapsular space of an immunodeficient SCID mouse. After transplantation, SCID mice were administered Jih1 via intravenous injection, and the spleens were extracted and used for antibody production. Figure 1A shows the antibody production scheme.

To measure the antigen specificity of the monoclonal antibodies, we screened the membrane fractions of Jih1 and *Bifidobacterium longum* JCM1217 by enzyme-linked immunosorbent assay (ELISA) and evaluated their reactivities against a broad panel of bacteria, including various bacterial taxa, as well as the target bacterium Jih1. For hybridoma screening, we selected clones that exhibited preferential reactivity toward Jih1 relative to other bacteria and retained proliferative capacity, ultimately selecting hybridoma clone 8H2. We then analyzed the reactivity of the 8H2 monoclonal antibody in the culture supernatant using fluorescence-activated cell sorting (FACS). The binding signal of 8H2 for some bacterial species was less than one-third of that observed for Jih1, which is consistent with preferential binding (Figure 1B). As such, we hypothesized that 8H2 could selectively bind to Jih1 cells to support enrichment from complex microbial communities. In addition, given that FACS analysis showed a strong signal (Figure 1B), we used 8H2 for immunofluorescence staining of Jih1. 8H2 successfully stained Jih1 but did not stain the IgG negative control (Figure 1C). These findings suggested that 8H2 could be used not only for FACS, but also for immunofluorescence staining. To further evaluate the applicability of SLOT beyond a single model bacterium, we also generated an antibody against *Phocaeicola dorei* (previously *Bacteroides dorei*), a Gram-negative bacterium isolated from a human fecal sample. Using the same approach, we were successfully able to produce an antibody that preferentially recognized *P. dorei* with limited reactivity toward other *Bacteroides* species (Supplementary Figure 2). This result suggests that SLOT can generate monoclonal antibodies exhibiting selective reactivity, supporting its potential for selective bacterial enrichment.

### Selective enrichment of Jih1 from complex gut bacterial communities using 8H2

**Figure 2.**
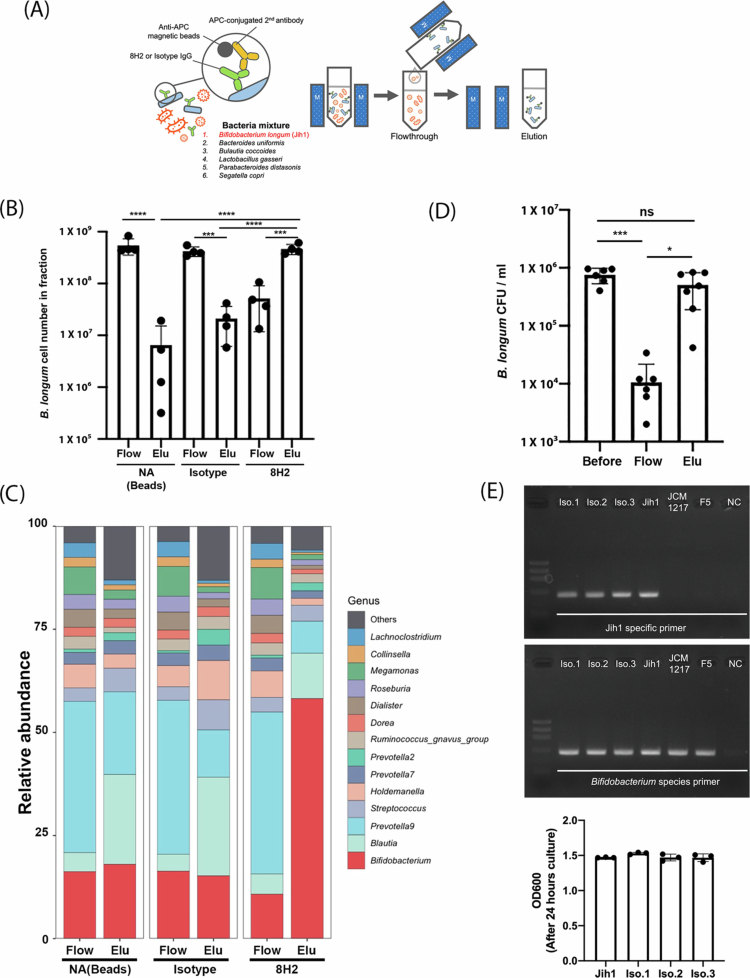
Selective enrichment of target bacteria from Jih1-containing bacterial mixture or Jih1-spiked fecal microbiota. (A) Schematic overview of *B. longum* Jih1 enrichment by MojoSort. (B) The bar chart shows *B. longum* cell number in each fraction. NA indicates samples incubated with anti-APC magnetic beads only. Significant differences between flow-through (Flow) and elution (Elu) were calculated using Tukey’s multiple comparisons test. ***P < 0.001. ****P < 0.0001. The data represent the mean values from four technical replicates using freshly prepared mixtures from the same bacterial stocks. (C) Bar chart showing the top 15 genus-level taxa and their relative abundances in the flow-through and elution fractions. The data represent the mean values obtained from three technical replicates, analyzed in a single 16S rRNA gene sequencing run. (D) Re-culturing of Jih1 after magnetic bead sorting from Jih1. The number of colonies is shown before sorting (before) and after sorting (Flow-through: Flow and Elution: Elu). Significances among fractions are shown by Tukey’s multiple comparisons test. *P < 0.05. ***P < 0.001. The data represent the mean values from six technical replicates. (E) Direct PCR using the bacterium culture supernatant. Isolation1-3, Iso.1–Iso.3; *B. longum* type strain 1217, JCM1217; human-derived *B. longum* isolate F5, F5; NC, negative control. The bar chart indicates OD600 after 24 hours of culture of each bacterium. The data represent the mean values from three technical replicates.

Next, we aimed to determine whether 8H2 could support the selective enrichment of Jih1 from a defined microbial consortium. To achieve this, we employed magnetic-activated cell sorting (MACS) as the separation method. MACS is an immunomagnetic separation technique in which antibody-bound target cells are captured using magnetic beads. MACS is a versatile method that can be performed under both aerobic and anaerobic conditions using compact instruments. Among various MACS platforms, we selected the Mojo device, a simple column-free system suitable for handling diverse bacterial samples. To evaluate performance and reproducibility, MACS was performed using independent technical replicates to process six bacterial solutions, which included typical gut bacterial species and the target bacterium Jih1 (Figure 2A). MACS was performed in each bacterial solution using magnetic beads alone, an isotype control antibody, or 8H2 (Figure 2A). Jih1 was detected at markedly higher levels in the 8H2-bound elution fractions than in the corresponding controls (Figure 2B), suggesting preferential and reproducible enrichment of Jih1 even in the presence of multiple gut bacterial species. This reproducibility is essential for demonstrating the practical applicability of the enrichment method. To further evaluate enrichment, we performed 16S ribosomal RNA (rRNA) gene analysis to assess whether Jih1 was enriched in the antibody-bound fraction. DNA was extracted from all the fractions separated by MACS and analyzed by 16S rRNA gene sequencing to determine the bacterial composition of each solution. Jih1 was approximately 3.9-fold more abundant in the 8H2 elution fraction compared with the IgG control elution fraction (Figure 2C), which is consistent with the selective enrichment of Jih1 from the defined bacterial mixture. We next examined whether the magnetic bead-bound bacteria would be culturable. A colony-forming unit (CFU) assay demonstrated that the magnetic bead‒antibody‒bacterium complexes were capable of forming colonies on agar plates (Figure 2D). Thus, the enrichment procedure preserved bacterial viability, supporting downstream applications requiring selective enrichment followed by cultivation of live bacteria. Furthermore, to identify characteristic bacteria in the elution fraction, we compared the bacterial compositions of the 8H2-bound elution and flow-through fractions using linear discriminant analysis effect size (LEfSe) analysis. The analysis revealed that *B. longum* was enriched in the 8H2 elution fractions, while *Phocaeicola* (formerly *Bacteroides*) and *Segatella (*formerly *Prevotella*) were more abundant in the flow-through fractions (Supplementary Figure 3). Finally, we tested whether 8H2 could enrich Jih1 directly from a human fecal sample without prior spiking. MACS using 8H2 resulted in increased relative abundance of Jih1, and the enriched bacteria formed colonies on selective agar plates (Figure 2E and Supplementary Figure 4). Taken together, these results suggest that 8H2 supports reproducible selective enrichment of viable Jih1 cells from both defined microbial consortia and human fecal samples.

### Identification of target molecules for 8H2

**Figure 3.**
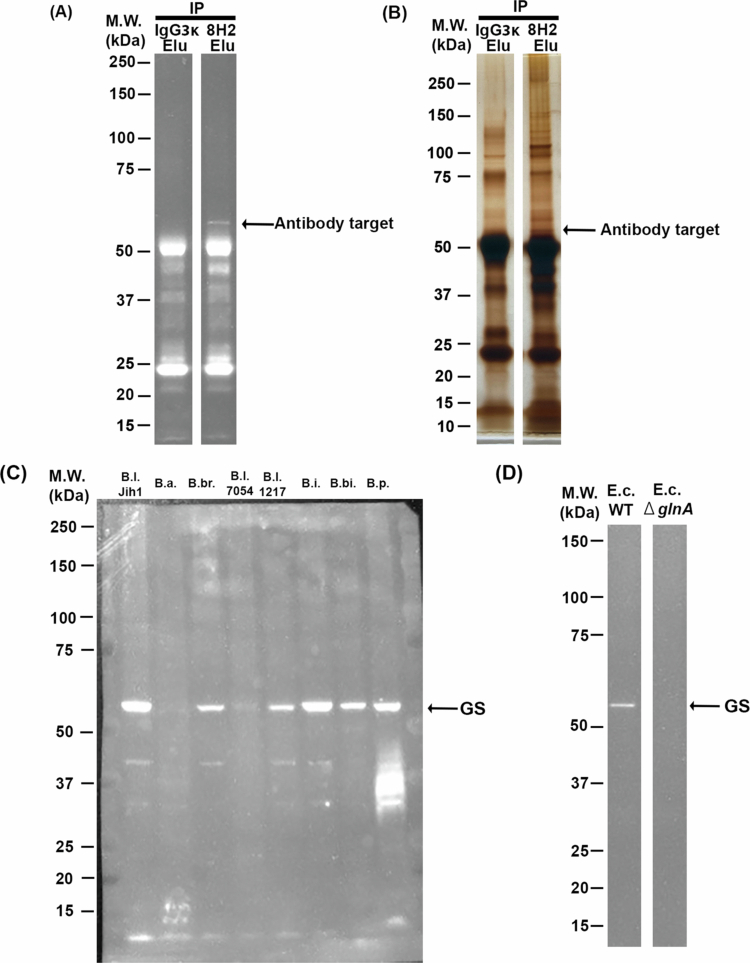
Identification of GS as the target antigen of 8H2. (A) Immunoprecipitation (IP) of the target protein was separated using IgG3κ and 8H2. The data are representative images from three technical replicates performed under identical conditions. (B) Silver staining of immunoprecipitation samples. The arrow shows the target protein. The data are representative images from three technical replicates performed under identical conditions. (C) Western blot detection of GS among *Bifidobacterium* species. B. l. Jih1: *B. longum* Jih1 strain, B.a.: *B. adolescentis, B.br.*: *B. breve*, B.l. 7054: *B. longum* JCM7054, B. l. 1217: *B. longum* JCM1217, B. i.: *B. infantis*, B. bi.: *B. bifidum* and B. p.: *B. pseudocatenulatum*. The arrow indicates glutamine synthetase (GS). The data shown are from one experiment in which all bacterial lysates were analyzed under identical conditions. (D) Western blot detection of GS in WT *E. coli* (E.c. WT) and *glnA* deletion *E. coli* (E. c. Δ*glnA*). The data are from one technical experiment.

To identify the Jih1 antigenic target recognized by the SLOT antibody, we first screened for candidate target molecules. Cell wall fractions from Jih1 were prepared and subjected to immunoprecipitation with 8H2 or an isotype control antibody. Immunoprecipitation revealed an approximately 55-kDa protein enriched in the 8H2-bound fraction (Figure 3A, B). This protein was subsequently isolated, purified, and identified by mass spectrometry as a type I glutamate-ammonia ligase, also known as glutamine synthetase (GS). GS, encoded by *glnA*, plays an important role in nitrogen metabolism and is conserved across various bacterial species.^26^ Specifically, GS catalyzes the ATP-dependent conversion of glutamate and ammonia to glutamine, a key step in cellular nitrogen assimilation. Within the genus *Bifidobacterium*, some genomes contain a *glnA* gene that has diverged evolutionarily and encodes a distinct amino acid sequence.^27^ We hypothesized that differences in the GS structure and/or expression would preclude certain bacteria from selection by 8H2, so we continued analysis of the GS protein to better understand the basis of its selective reactivity.

**Figure 4.**
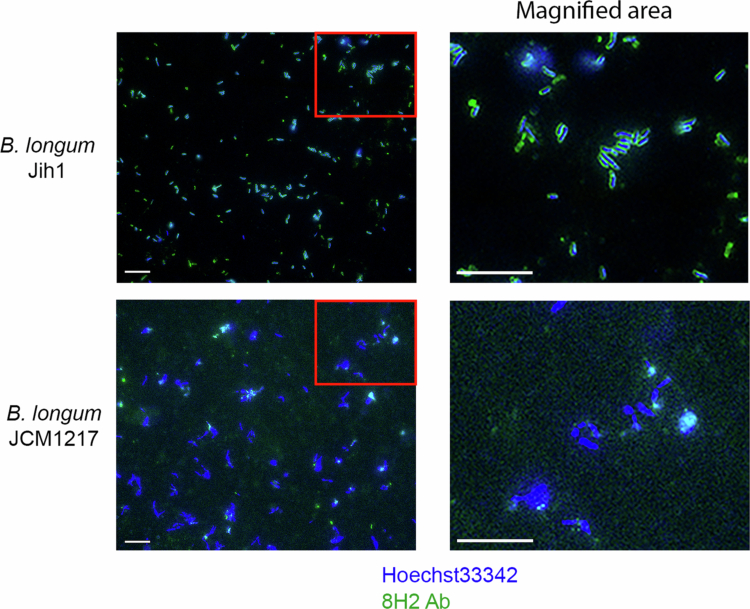
Comparison of GS expression patterns between the Jih1 isolate and JCM 1217 type strain. Immunofluorescence staining of *B. longum* Jih1 (upper) and *B. longum* JCM1217 (lower). The right panel shows an enlarged view of the left panel. *B. longum* cells stained with Hoechst 33342 are fluorescent blue, and 8H2 antibodies are fluorescent green. The red box indicates the area of enlargement shown on the right. Scale bars, 10 μm. Representative images from at least three technical replicates.

We evaluated the expression of GS among different *Bifidobacterium* species by Western blotting of bacterial lysates and found that, although GS was commonly expressed among the tested species, the expression levels varied (Figure 3C). Interestingly, sequence analysis indicated that GS is highly conserved among closely related *Bifidobacterium* species, exhibiting over 90% identity (Supplementary Figure 5), and shared approximately 50% amino acid identity with *Escherichia coli* GS (Supplementary Figure 5). We tested the 8H2 reactivity to *E. coli*-derived GS by performing western blotting using *glnA*-deleted *E. coli* mutants. For the wild-type (WT) strain, we selected *E. coli* BW25113, the parent strain of the Keio collection of gene deletion mutants, owing to its well-characterized genetic background and suitability for reproducible experiments.^28^ Western blot analysis showed that no signal was detected in the *glnA*-deleted *E. coli*, whereas a signal was detected in WT *E. coli* (Figure 3D), supporting the conclusion that GS was indeed the antigen recognized by 8H2 (Figure 3C,3D). However, it was unclear how such a widely conserved, intracellular enzyme^29^ could be the target of 8H2, which supported selective enrichment of Jih1 from a fecal microbial consortium. Further investigation revealed that Jih1 was detectable via 8H2 binding in FACS (Figure 1B), suggesting that GS may be accessible on the cell surface; immunofluorescence staining of *B. longum* indeed revealed GS expression on the surface of Jih1 cells. Notably, this surface expression pattern was not evident in other human-derived *B. longum*, and signs of cell-surface staining were limited in these bacteria (Figure 4). Collectively, these findings suggest that Jih1 may exhibit a distinct pattern of GS localization, which may help explain the preferential reactivity of 8H2 and the observed enrichment of Jih1 within complex microbial communities.

## Discussion

In this study, we generated monoclonal antibodies exhibiting selective reactivity toward a target bacterium using SLOT (Figure 1, Supplementary Figure 2). Since SLOT has not previously been applied to whole bacterial cells as a target, our results suggest that this approach is capable of generating monoclonal antibodies exhibiting defined antigen-binding characteristics and preferential reactivity toward selected bacteria. Notably, monoclonal antibodies that cross-reacted with multiple taxa other than Jih1 were excluded during the screening process. We also obtained a monoclonal antibody against *P. dorei* that showed no detectable cross-reactivity with closely related *Bacteroide*s subspecies or *Parabacteroides distasonis* under the tested conditions (Supplementary Figure 2). In the context of artificial lymph node transplantation, it has been shown that antigen-specific antibody-forming cells, germinal center B cells, and memory B cells are enriched.^24^ A similar immune mechanism may have contributed to the generation of the preferentially reactive antibodies observed in this study.

Through further application of SLOT, we developed a method for enriching target human-derived bacteria using monoclonal antibodies exhibiting selective reactivity (Figures 1A–C and 2A–D). The 8H2 monoclonal antibody was able to preferentially react with Jih1 relative to other tested bacteria. Notably, 8H2 exhibited approximately 20.7 ± 2.9% reactivity with *Bifidobacterium bifidum*, *Segatella copri*, and *Lactobacillus gasseri* (Figure 1B), suggesting that 8H2 may recognize shared physicochemical features on membrane surface sites of these bacteria. However, these cross-reactive bacterial species were not detected in the MACS elution fractions, which is consistent with the selective enrichment of the target bacterium from both a defined bacterial consortium and a human fecal sample (Figure 2C,E). Thus, the observed level of cross-reactivity appeared compatible with the enrichment procedure used in this study. Furthermore, the enriched bacteria remained viable and re-culturable (Figure 2D,E), indicating that this approach may support selective enrichment followed by cultivation of live bacteria from the human fecal microbiota. Taken together, this method may support personalized microbiome research by enabling access to viable, selectively enriched individual-associated bacteria from complex microbial communities.

Amino acid sequence divergence alone is unlikely to fully explain the observed extracellular localization in Jih1, as shown in our comparative analysis of the amino acid sequences of Jih1 glnA and those of closely related species (Supplementary Figure 5). Unlike the wild-type *B. longum* strain, which exhibited both internal and external staining, Jih1 showed predominantly extracellular GS localization (Figure 4). As shown in Figure 3D, no signal was detected in the *glnA* deletion mutant, supporting that the antibody recognizes GS under the test conditions. GS is traditionally known as an enzyme essential for nitrogen metabolism, catalyzing the synthesis of glutamine by condensation of glutamate and ammonia.^30^ Despite this canonical intracellular role, Jih1 exhibited clear surface-associated GS signals. Although the mechanism underlying GS exposure in Jih1 remains unknown, these observations support that GS surface localization may represent a phenotypic feature observed in Jih1, and suggest that GS is an antigen associated with the preferential reactivity of the SLOT-derived monoclonal antibody.

Previous studies have not reported the identification of target antigens that enable selective enrichment in the context of closely related bacteria.^10^^,^^16^ Our study identified GS as an antigen associated with preferential antibody reactivity in Jih1, supporting the feasibility of selective enrichment under the tested conditions. However, it must be noted that strain-level selectivity has not been verified, and results may vary based on the context in which these antibodies are used. For instance, our GS-based 8H2 antibody may fail to enrich *B. longum* Jih1 in environments that include other bacteria exhibiting cell surface-localized GS. We hypothesize that our method is best suited for intra-individual or longitudinal studies in which antibody selectivity can be contextually verified. Such selectively reactive antibodies may provide a useful tool for monitoring the persistence and dynamics of individual-associated bacteria within the same host over time, thereby supporting longitudinal analyses of intra-individual microbiome variation. In contrast, our study identified a distinct antigen and a unique GS expression pattern in Jih1, providing proof-of-concept support for the selective enrichment of target bacteria by our method.

Future studies focusing on bacteria that exhibit surface-associated or differentially expressed proteins may facilitate selective enrichment through antibody-based approaches targeting such features. Given that this approach successfully generated a selectively reactive monoclonal antibody against *P. dorei*, it may be applicable to a broader range of gut bacteria. However, we emphasize that additional studies across more taxa will be required to fully define its scope and limitations. Altogether, our study provides a practical proof-of-concept for monoclonal antibody-based selective enrichment of target bacteria from complex microbial communities. This methodology may contribute to personalized microbiome research by enabling the selective enrichment and monitoring of individual-associated bacteria as well as facilitating downstream molecular and functional analyses.

## Methods

### Animal experiment

Animals were purchased from CLEA Japan and maintained in the Keio University animal facility. Germ-free mice were bred and raised in axenic isolators at the Tsukuba University animal facility. The animal experimental protocols were approved by the Institutional Animal Care and Use Committee of Keio University and the University of Tsukuba.

### Bacteria culture

A list of bacteria used in this study is shown in Table 1. *Bifidobacterium longum* Jih1 (Jih1), F5, Isolation 1, Isolation 2, and Isolation 3 strains were isolated from a healthy person. Other type strains were purchased from the RIKEN BRC Japan Collection of Microorganisms (JCM). These cells were grown in 5.0 mL of liquid Gifu Anaerobic Medium broth (GAM) (Nissui, Japan) for 24–48 h at 37 °C in a BACTRON 300 anaerobic chamber (Shel Lab, USA). Cultured bacteria were harvested by centrifugation at 4400 g for 15 min at 4 °C. Bacterial pellets were washed with sterile 1 × Phosphate-Buffered Saline (PBS) twice and stored at –80 °C until the experiment.

**Table 1.** Bacterial strains used in this study.

Bacteria name	Strain	Source
*Bifidobacterium longum*	Jih1^a^	Human
*Bifidobacterium longum*	F5^a^	Human
*Bifidobacterium longum*	JCM1217^b^	Human
*Bifidobacterium bifidum*	JCM7004^b^	Human
*Bifidobacterium breve*	JCM1192^b^	Human
*Bifidobacterium infantis*	JCM1222^b^	Human
*Bifidobacterium adolescentis*	JCM1275^b^	Human
*Bifidobacterium animalis*	JCM1253^b^	Chicken
*Bifidobacterium pseudocatenulatum*	JCM1200^b^	Human
*Bifidobacterium pseudolongum*	JCM1205^b^	Swine
*Parabacteroides distasonis*	JCM5825^b^	Human
*Segatella copri*	JCM13464^b^	Human
*Bacteroides uniformis*	JCM5828^b^	Human
*Blautia obeum*	JCM31340^b^	Human
*Lacticaseibacillus rhamnosus*	JCM1136^b^	Human
*Lacticaseibacillus casei*	JCM1134^b^	Cheese
*Lactobacillus gasseri*	JCM1131^b^	Human
*Escherichia coli*	JCM1649^b^	Urine
*Blautia coccoides*	JCM1395^b^	Mouse
*Phocaeicola dorei*	JCM13471^b^	Human
*Bacteroides ovatus*	JCM5824^b^	Human
*Bacteroides stercoris*	JCM9496^b^	Human
*Bacteroides thetaiotaomicron*	JCM5827^b^	Human
*Phocaeicola vulgatus*	JCM5826^b^	Human
*Bifidobacterium longum*	Isolation1 (Iso1)^a^	Human
*Bifidobacterium longum*	Isolation2 (Iso2)^a^	Human
*Bifidobacterium longum*	Isolation3 (Iso3)^a^	Human

^a^ Isolated from healthy Japanese in this study.

^b^ Purchased from Japan Collection of Microorganisms (JCM).

### Secondary lymphoid organ transplantation (SLOT)

The cultured Jih1 was harvested by centrifugation at 4400 g for 15 min at 4°C. The bacterial pellets were washed with 5.0 mL of sterile PBS, followed by centrifugation at 4400 g for 5 min at 4 °C. Two more washes were performed afterward. The bacterial pellets were dried for 24 h by VD-800R lyophilizer (TAITEC, Japan). The female 8-week-old BALB/cAJcl mice were purchased from CLEA Japan, Inc. All mice (*n* = 5) were immunized with 100 μg of whole bacteria (1.0 mg/mL) with complete Freund adjuvant (Thermo Scientific, USA) via intraperitoneal (i.p.) injection twice every two weeks. The spleens were harvested from two immunized mice and minced. Small pieces of the spleen were transplanted under the fibrous capsule of the kidney from 8-week-old female FOX CHASE SCID CB-17/1cr-scid/scid (CLEA, Japan) (SCID) mice (*n* = 3). The other three immunized BALB/cAJcl (BALB/c) mice were controlled, not treated with SLOT. A week after transplantation, the transplant-recipient mice and control mice were further immunized with 100  μg of whole bacteria diluted with sterile PBS via intravenous (i.v.) injection five times every other week. The establishment of hybridomas and the purification of antibodies were performed by BioGate Co., Ltd.

### Bacteria fluorescence-activated cell sorting (FACS)

The glycerol stock of each bacterial strain was cultured in GAM liquid medium for 24 h at 37 °C under anaerobic conditions. The bacterial suspension was centrifuged at 4400 × g for 5 min at 4 °C, and the supernatant was removed. The 1 × 10^7^ bacteria were blocked with 2.0% (w/v) BSA/PBS for 30 min at 4 °C. The suspension was centrifuged at 17,800 g for 5 min at 4 °C. These bacterial pellet samples were suspended with 100.0 µL of the hybridoma cell culture supernatant for 30 min at 4 °C. Mouse anti-IgG3 isotype control (MG3-35, BioLegend) was diluted with 2.0% BSA/PBS and used as a negative control. For screening of *P. dorei*, a mouse anti-IgG2a isotype control (MG2a-53, BioLegend) was used. After the samples were washed and incubated with 20 μg/ml goat Anti-mouse IgG secondary antibody [FITC] (Abcam) for 30  min at 4 °C. The samples were resuspended in 400  µL with sterile 1 ×  PBS to analyze on Accuri C6 Plus (BD Bioscience). The bacterial populations were selected based on their FSC-SSC values. The proportion of FITC-positive cells within this gate was defined as the antibody reaction rate. The isotype control-stained population was used as a background for each comparison.

### Preparation of the six-bacterial mixture and the human microbiota suspension

The six-bacterial mixture was comprised of Jih1, *B. coccoides*, *L. gasseri, S. copri*, *P. distasonis*, and *B. uniformis*. These bacteria were adjusted to 1.0 × 10^7^ cells with sterile PBS containing 2.0% (w/v) BSA (Jackson ImmunoResearch, USA) and mixed at equal volumes. The total amount of the six bacterial mixtures was 6.0 × 10^7^ cells. For preparation of the defined microbial consortium, a human gut microbiota solution was prepared by filtering out the narrow matter from the human fecal suspension. The target bacterium Jih1 (OD = 0.1) was spiked into the gut bacterial solution (OD = 0.5), thus resulting in Jih1 accounting for approximately 16.7% of the total solution. The fecal suspension was filtered using 1000, 500, 100, 40, and 20 μm filters (pluriStrainer, pluriSelect, Germany) for removing undigested food. The human microbiota was suspended in sterile PBS containing 2.0% (w/v) BSA (Jackson ImmunoResearch, USA). Then, 1.0 × 10^7^ of Jih1 cells were added to 8.0 × 10^7^ human microbiota cells.

### Target bacteria enrichment by MojoSort

The six-bacteria mixture or Jih1-spiked human microbiota suspension was blocked with 2.0%BSA/PBS for 15  min at room temperature. The samples were incubated with 10  μg/mL of 8H2 or purified mouse IgG3 Isotype Ctrl antibody (BioLegend, USA) in 2.0%BSA/PBS for 30  min at 4 °C , and then washed with sterile PBS three times. Afterward, the samples were incubated with Goat anti-mouse IgG Cross-Adsorbed secondary antibody, APC (2.0  μg/mL: Thermo Fisher Scientific, USA) in 2.0%BSA/PBS for 30  min and then washed with sterile PBS three times. The bacterial pellets were dissolved with MojoSort Buffer (BioLegend, USA), and 10.0 µL of MojoSort mouse anti-APC Nanobeads (BioLegend, USA) were added before incubating for 15  min at 4 °C. The samples were washed with sterile PBS three times and resuspended in MojoSort Buffer. They were then placed in the MojoSort magnet (BioLegend, USA) for 5 min at room temperature before being poured out, and the fraction was collected as a flow-through. This process was repeated twice, after which the tube was removed from the magnet. Both the Flow-through and Elution fraction tubes were centrifuged at 17,800 g, for 5 min at 4 °C. The pellets were dissolved with sterile water or PBS. After collecting the pellets, DNA was extracted from each solution using the respective pellets, followed by qPCR and 16S rRNA gene sequencing.

### DNA extraction

DNA samples were extracted from the bacterial pellets. DNA extraction was performed as described in a previous study, with some modifications.^31^ In brief, the samples were first incubated with 15.0 mg/mL lysozyme (Wako Co., Japan) at 37 °C overnight. Next, the lysates were further incubated with achromopeptidase (Wako Co., Japan) at a final concentration of 600 units/mL at 37 °C for 8  h. Then, SDS and Proteinase K (Merck Millipore Ltd., Germany) were added to reach final concentrations of 1.0% and 1.0 mg/mL, respectively, and the samples were incubated at 55 °C overnight. After that, bacterial genomic DNA was purified from each sample according to the standard phenol-chloroform/isoamyl alcohol protocol as described in a previous study.^32^

### Quantitative PCR

Each PCR mixture (20.0 µL) was composed of 10.0 µL of SYBR Premix Ex Taq Ⅱ (Takara, Japan), 0.4 µL of ROX Reference Dye (50×), 0.8 µL of species-specific primer at a concentration of 10.0 µM, 6.0 µL of ultrapure water, and 2.0 µL of template DNA at a concentration of 10.0 ng/µL. The abundance of *Phocaeicola* (*previously Bacteroides*), *Segatella* (previously *Prevotella*), *P. distasonis*, and *B. longum* were estimated to use bacteria-specific primers (Table 2) with a Step-One Real-Time PCR system (Applied Biosystems, USA). The following amplification program was used: one cycle consisting of 95 °C for 30 s, followed by 40 cycles consisting of 95 °C for 5 s, 60 °C for 60 s, and finally 3 steps by 95 °C for 15 s, 60°C for 1 min, and 95 °C for 15 s. The abundances of *B. longum*, was calculated by the relative abundance of the 16S rRNA gene (Table 2) copy number.

**Table 2.** Quantitative PCR and PCR primers used in this study.

Target	Direction	Sequence (5'–3')	Reference
16S rRNA genes	Forward	AGRGTTTGATYMTGGCTCAG	[8]
	Reverse	TGCTGCCTCCCGTAGGAGT	[8]
*Prevotella*	Forward	CACCAAGGCGACGATCA	[33]
	Reverse	GGATAACGCCYGGACCT	[33]
*Bacteroides*	Forward	GAGAGGAAGGTCCCCCAC	[34]
	Reverse	CGCTACTTGGCTGGTTCAG	[34]
*Bifidobacterium longum*	Forward	TGGAAGACGTCGTTGGCTTT	[35]
	Reverse	ATCGCGCCAGGCAAAA	[35]
*Parabacteroides distasonis*	Forward	TGATCCCTTGTGCTGCT	[36]
	Reverse	ATCCCCCTCATTCGGA	[36]
*Bifidobacterium longum* Jih1	Forward	AACGAGGCAGGCAACACTAA	This study
	Reverse	TGTAGTCGGTCGTGTATTGTCC	This study
*Bifidobacterium longum* JCM1217	Forward	AGTCAAGAACATCACCAACCTG	This study
	Reverse	AGATTGACTGCCTTGCGAC	This study

## 16S rRNA gene sequencing

The V1–V2 variable regions of the 16S rRNA gene were amplified using the bacterial universal primers 27F-mod (5′-AGRGTTTGATYMTGGCTCAG-3′)^8^ and 338R (5′-TGCTGCCTCCCGTAGGAGT-3′)^8^ with Tks Gflex DNA Polymerase (Takara, Japan). Amplicon DNA was sequenced using MiSeq (Illumina, USA) according to the manufacturer’s protocol. The sequenced reads were processed using the Quantitative Insights into Microbial Ecology 2 (QIIME 2, version 2019.7)^37^ with DADA2 to generate amplicon sequence variants (ASVs). Taxonomic assignment was performed using the SILVA 132 database. The minimum read depth across samples was 10,568 reads per sample.

### LEfSe analysis

A linear discriminant analysis (LDA) effect size (LEfSe) tool (http://huttenhower.sph.harvard.edu/lefse/) was used to identify differentially abundant taxa between the elution fraction and flow-through fraction. A non-parametric Kruskal-Wallis test and unpaired Wilcoxon rank sum test allowed for the identification of differentially abundant taxa among sample groups and the LDA method was used to estimate the effect size of each feature/taxa within a given group.^38^ LEfSe analysis was performed with the alpha value for statistical analyses set to 0.05 and the threshold on the logarithmic LDA score for discriminative features set to 5.0.

### Selective enrichment of Jih1 from a human fecal sample

MojoSort was performed with the gut bacterial solution (OD = 1.0) and 8H2 antibody. The target fractions were seeded onto TOS Propionate Agar Medium (Yakuruto Pharmaceutical Industry, Japan) and allowed to form colonies. Colonies were picked up and cultured in GAM liquid medium, and OD600 measurement was performed. Bacterial pellets were collected by centrifugation and washed with PBS. Resuspended in water or PBS, the pellet was used for PCR and Gram staining. PCR with Jih1-specific and *Bifidobacterium* species primers (Table 3) was performed using Tks Gflex DNA Polymerase (Takara, Japan) with the following amplification program: one cycle consisting of 95 °C for 5  min, followed by 25 cycles consisting of 98 °C for 10 s, 56 °C for 15 s, and 68 °C for 30 s, finally, 68 °C for 3  min, after which samples were held at 4 °C.

**Table 3.** PCR primers used in this study.

Target	Direction	Sequence (5'–3')	Reference
*Bifidobacterium longum* Jih1	Forward	CGGACGAATGGACTACGACA	This study
	Reverse	TTGTGGCTCTTCGTGTCCTC	This study
*Bifidobacterium species*	Forward	CTCCTGGAAACGGGTGG	[39]
	Reverse	GGTGTTCTTCCCGATATCTACA	[39]

### Gram staining

A bacterial smear was heat-fixed onto a slide. Crystal violet was added for 1 min and rinsed with water. Add iodine solution for 30 s and rinse with water. Repeat this process once more. Add ethanol–acetone for decolorization and rinse with water. A fuchsin-based solution was applied for 1 min and rinsed with water to counterstain. The slide is dried and examined under a microscope using oil immersion BX53 (OLYMPUS, Japan).

### Immunoprecipitation

Jih1 cell wall proteins were extracted from 90 mL of bacterial culture pellet according to a previously reported method (Candela M, et al. J Bacteriol. 2007. PMID: 17557824). Briefly, Jih1 was collected by centrifuging at 5000 rpm for 10 min at 4 °C. The bacterial pellet was washed with 50 mM Tris-HCl (pH 7.6) twice. It was then resuspended with 2 mL of buffer containing 50 mM Tris-HCl (pH 7.6)/1 M sucrose/15 mg/mL lysozyme/complete inhibitor EDTA-free (Roche, Austria) and incubated for 90  min at 4 °C. Cell wall proteins were collected by centrifugation at 4000  rpm for 3  min at 4 °C. The samples were stored at –80 °C until the experiment. The cell wall proteins were incubated with Protein A Dynabeads and 10 μg 8H2 antibody in PBS-T overnight at 4 °C with rotation. The immunoprecipitates were recovered, washed, resolved in NuPAGE 4%–12.0% Bis–Tris gels (Novex, USA), and immunoblotted with 8H2 antibody isotype control IgG. Proteins of interest were visualized using HRP-conjugated secondary Ab (Jackson ImmunoResearch, USA) and enhanced with SuperSignal West Dura extended duration substrate (Thermo Fisher, USA).

### Mass spectrometry

Immunoprecipitate sample proteins were electrophoresed in NuPAGE 4%–12.0% Bis–Tris gels run in MOPS buffer (Invitrogen, USA). Proteins in the SDS gel were stained using Silver Stain KANTO III (Kanto, Japan) according to the manufacturer's instructions. The targeted bands were cut from the gel and mass spectrometry was performed at IDEA (IDEA consultants, Inc., Japan).

### Western blotting

*Bifidobacterium* species and *E. coli* were cultured in GAM medium under anaerobic conditions. Bacteria in PBS containing complete protease inhibitor without EDTA (Roche) were homogenized via sonication (TOMY, Japan) or mechanical disruption by glass beads (Shakemaster, Bio Medical Science Inc., Japan). Protein concentrations were determined by BCA protein assay (Takara, Japan), and normalized amounts of protein were denatured in 6X SDS sample buffer (Nacalaitesque, Japan) at 95 °C for 10 min. Two or 10 μg of bacterial protein was electrophoresed in NuPAGE 4%–12.0% Bis–Tris gels run in MOPS buffer (Invitrogen, USA), transferred onto a PVDF membrane, and blocked with 5% skim milk (Wako, Japan) in PBS-T. The membrane was incubated with 2  μg/mL 8H2 (this study) or isotype control IgG overnight at 4 °C. PBST-washed blots were incubated for 1 h at R.T. with 0.5  μg/mL horseradish peroxidase-conjugated secondary Ab (Invitrogen, USA), washed, and exposed to SuperSignal West Dura (Thermo Fisher Scientific, USA) extended duration substrate. Proteins of interest were detected with a chemiluminescent detection system (BioTools MISVS II, BioTools, Japan).

### Immunofluorescence microscopy

Bacteria were fixed with 10% Formalin Solution (Wako) for 15 min at RT and rinsed with PBS three times. Specimens were placed on a glass slide (Matusnami, Japan) and dried thoroughly. Further fixation was done using methanol (Wako, Japan), and specimens were dried thoroughly before blocking with 2.0% BSA/PBS for 15 min and washing with PBS-T three times. The samples were then incubated with 5 µg/mL of 8H2 or Purified mouse IgG3 Isotype Ctrl antibody (BioLegend, USA) in 2.0% BSA/PBS for 30 min and washed with PBS. Then, the samples were incubated with Goat anti-mouse IgG Alexa Fluor 488 (2  μg/mL: Thermo Fisher Scientific, USA) in 2.0% BSA/PBS for 30  min and washed with PBS. Bacteria were counter-stained with Hoechst 33342 (Thermo Fisher Scientific, USA). After washing with PBS, the cells were covered with a cover glass and observed under a fluorescence microscope. Fluorescence microscopic images were obtained using a BX53 (OLYMPUS, Japan) and Mosaic 2.4 software (BioTools, Japan). ImageJ was used for image analysis.

### *In vivo* competition assay

For the competition assay between Jih1 and *B. longum* JCM 1217, male and female BALB/c GF mice were fed a normal diet in axenic isolators. Jih1 and *B. longum* JCM 1217 were grown at 37 °C in GAM medium under anaerobic conditions, diluted to OD = 1.0/mL in GAM medium, and mixed at a 1:1 ratio. Bacterial suspensions were administered orally to germ-free mice. Murine feces were collected on Day 0 and after 1 week, 2 weeks, 3 weeks, and 5 weeks. Fecal pellets were stored at –80 °C until the experiment. Fecal DNA from the mice was extracted using ISOPLANT II (NIPPON GENE, Japan) according to the manufacturer’s instructions. The amount of DNA present in each *B. longum* strains was determined by qPCR using strain-specific primer set (Table 2).

### Membrane vesicle purification and quantification

Jih1 and *B. longum* JCM 1217 were cultured in 25  mL of MRS (BD, USA) medium at 37 °C for 24  h under anaerobic conditions (MGC, Japan). Culture supernatants were separated by centrifugation at 6000 g for 20 min at 4 °C and were filtered through 0.45-μm-pore-sized polyvinylidene difluoride (PVDF) filter membranes (Millipore, Germany). The filtered supernatants were ultracentrifuged at 200,000 g for 1 h at 4 °C, and the pellets were washed with PBS. The samples were ultracentrifuged again at 200,000 g for 1 h at 4 °C, and the pellets were diluted with 100.0  μL of pure water. MVs were stained with 2.5 μM FM1-43 (Invitrogen, USA) and quantified with a fluorescent plate reader (Synergy H1 BioTek, Agilent, USA).

### *glnA* homology analysis

The amino acid sequences of *glnA1* were extracted from *Bifidobacterium* genomes, *B. animalis* subsp. *animalis* ATCC 25527 (NC_017834.1), *B. bifidum* JCM 1255 (NZ_AP012323.1), *B. breve* JCM 1192 (NZ_ACCG00000000.2), *B. longum* subsp. infantis JCM 1222 (AP010889.1), *B. longum* subsp. *longum* JCM 1217 (AP010888.1), *B. longum* subsp. *longum* JCM 7052 (AP022379.1), and *B. pseudolongum* ATCC25526 (NZ_CP093555.1). Extracted *glnA1* sequences and *glnA* from *E. coli* BW25113 (NZ_CP009273.1) were used for the MAFFT multiple alignment program (v7.490) as implemented in Geneious Prime 2023.1.2.

### Statistical analysis

Statistical analysis was performed in R version 4.2.2 or Prism software version 8.4.3 (GraphPad). Significance metrics for the reactivity of the hybridoma culture supernatant and membrane vesicle production were calculated using an unpaired one-tailed t-test with Welch correction. Significant differences between fractions (flow-through, elution, and pre-enrichment) were calculated using Tukey’s multiple comparisons test. In this study, biological replicates refer to independently treated animals, whereas technical replicates refer to repeated measurements performed on samples prepared from the same experimental conditions. Unless otherwise stated, the experiments were performed using technical replicates. The type and number of replicates used in each experiment are specified in the corresponding figure legends.

## Supplementary Material

Supplementary Material KGMR-2025-0062.R3 Supplemental_data.docx

## Data Availability

The 16S rRNA gene amplicon sequence files generated in this study are available in the DNA Data Bank of Japan (DDBJ) as PRJDB17673. The data of this study are available from the corresponding author [S.F.] upon reasonable request.
