# Supplementary material for: Development of a monoclonal antibody-based approach for selective enrichment of target Bifidobacterium longum from a complex fecal community
Source: Gut Microbes Rep. 2026 Apr 29;3(1):2663732. doi: 10.1080/29933935.2026.2663732 (PMC13134421; doi:10.1080/29933935.2026.2663732)
Supplement: Supplementary Material — KGMR-2025-0062.R3 Supplemental_data.docx [file KGMR_A_2663732_SM4897.docx]

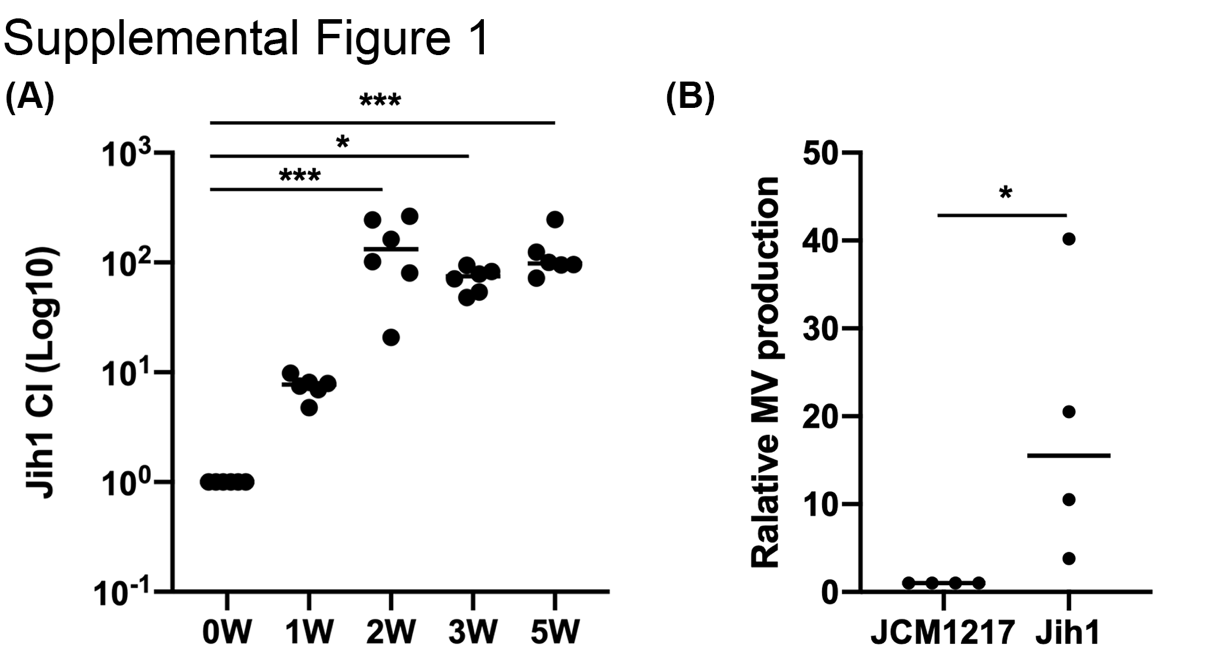


Supplemental Figure 1 Features of *B. longum* Jih1 strain

(A) *in vivo* competition assay between *B. longum* Jih1 strain (Jih1) and *B. longum* JCM1217 (JCM1217). The log10 competition index (DNA amount of Jih1 divided that of JCM1217) is shown as calculated by Dunn’s multiple comparisons test. **P* < 0.05 ****P* < 0.001. Data represent biological replicates from six independently treated mice. (B) Membrane vesicle (MV) production between Jih1 strain and JCM1217. Significances between Jih1 and *B. longum* JCM1217 are calculated by Mann-Whitney test. *P < 0.05. Data represent mean from four independent technical experiments.


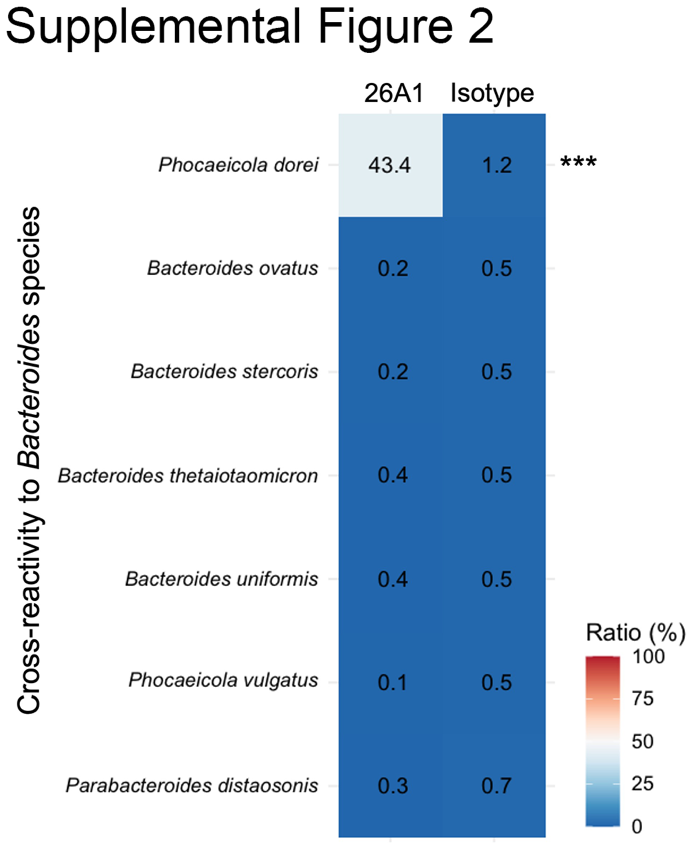


Supplemental Figure 2 Generation of human *Phocaeicola dorei*-specific antibody

Reactivity of anti-*Phocaeicola dorei* monoclonal antibody 26A1. Heatmap of reactivity against single gut bacteria by flow cytometry. Significances between the reactivity of IgG2a isotype control antibody and each bacterium are shown by Welch’s t-test. ***P* < 0.01, ****P* < 0.001. Data represent mean value from three technical replicates.


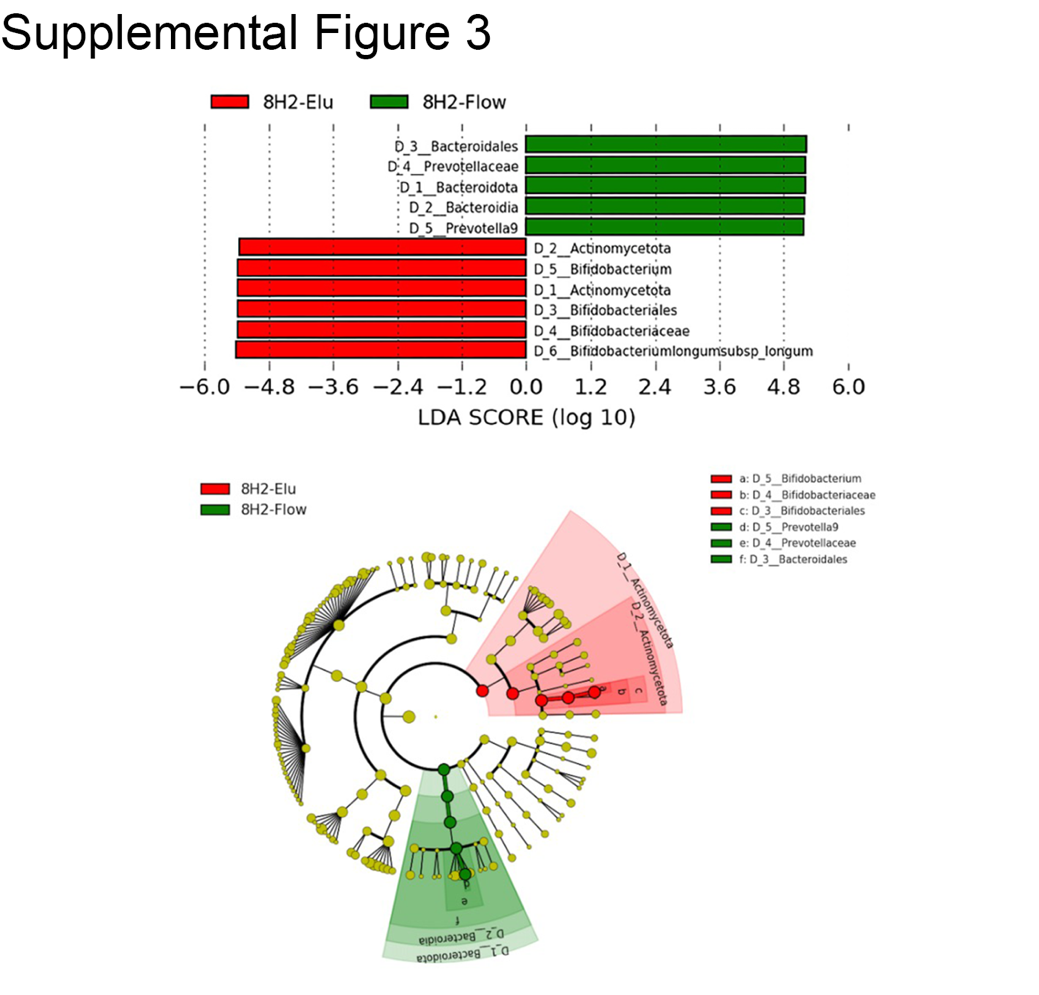


Supplemental Figure 3 LEfSe analysis of bacteria isolated by 8H2 in fractions taken from human intestinal microbiota spiked with Jih1.

The bar plots represent the significantly differential taxa in each bacterial level between Flow through (green) and Elution (red) separated utilizing 8H2, based on effect size (LDA score [log 10]. The cladogram shows significantly different taxa in Flow through (green) and Elution (red).


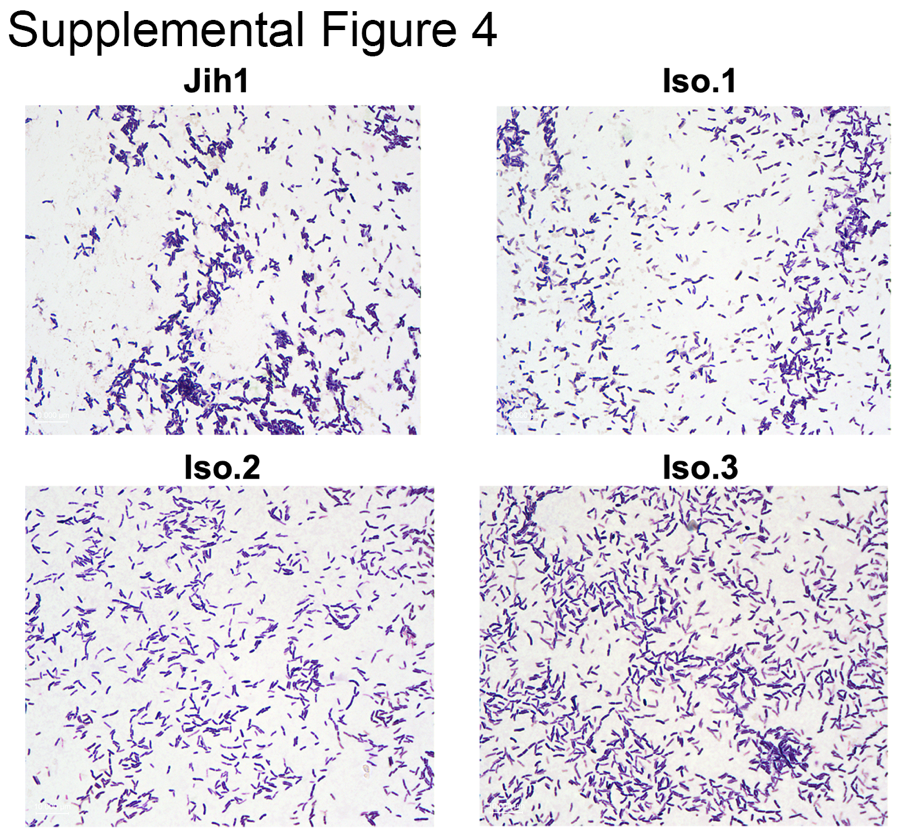


Supplemental Figure 4 Gram staining of isolated Jih1 strain from the original fecal sample

Gram-stained image of each bacterium after cultivation in GAM liquid medium. Representative images from at least three technical replicates.


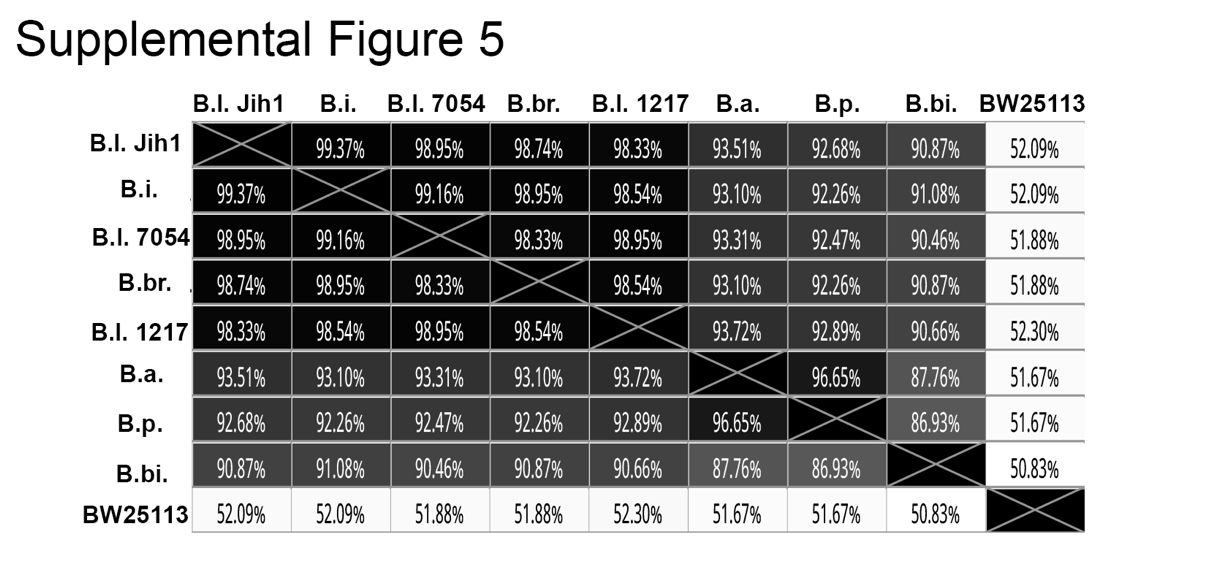


Supplemental Figure 5 Homology analysis of amino acid sequences of *glnA* among *Bifidobacterium* species and *Escherichia coli.*

More than 90% of amino acid sequences were shared among *Bifidobacterium* species. Only 50% of *glnA* amino acid sequence was preserved between *Bifidobacterium* species and *E. coli*.
